# Cdk5 Modulates Long-Term Synaptic Plasticity and Motor Learning in Dorsolateral Striatum

**DOI:** 10.1038/srep29812

**Published:** 2016-07-22

**Authors:** Adan Hernandez, Chunfeng Tan, Gabriel Mettlach, Karine Pozo, Florian Plattner, James A. Bibb

**Affiliations:** 1 Departments of Psychiatry, Neurology and Neurotherapeutics and Harold C. Simmons Comprehensive Cancer Center, The University of Texas Southwestern Medical Center, Dallas, TX 75390, USA

## Abstract

The striatum controls multiple cognitive aspects including motivation, reward perception, decision-making and motor planning. In particular, the dorsolateral striatum contributes to motor learning. Here we define an approach for investigating synaptic plasticity in mouse dorsolateral cortico-striatal circuitry and interrogate the relative contributions of neurotransmitter receptors and intracellular signaling components. Consistent with previous studies, we show that long-term potentiation (LTP) in cortico-striatal circuitry is facilitated by dopamine, and requires activation of D1-dopamine receptors, as well as NMDA receptors (NMDAR) and their calcium-dependent downstream effectors, including CaMKII. Moreover, we assessed the contribution of the protein kinase Cdk5, a key neuronal signaling molecule, in cortico-striatal LTP. Pharmacological Cdk5 inhibition, brain-wide Cdk5 conditional knockout, or viral-mediated dorsolateral striatal-specific loss of Cdk5 all impaired dopamine-facilitated LTP or D1-dopamine receptor-facilitated LTP. Selective loss of Cdk5 in dorsolateral striatum increased locomotor activity and attenuated motor learning. Taken together, we report an approach for studying synaptic plasticity in mouse dorsolateral striatum and critically implicate D1-dopamine receptor, NMDAR, Cdk5, and CaMKII in cortico-striatal plasticity. Furthermore, we associate striatal plasticity deficits with effects upon behaviors mediated by this circuitry. This approach should prove useful for the study of the molecular basis of plasticity in the dorsolateral striatum.

Striatal circuitry mediates procedural or implicit learning that results in automatized responses, roughly equivalent to habits^1^,^2^,^3^. Dorsolateral striatal neurons change their activity during procedural learning tasks in mice^4^, rats^5^,^6^,^7^, and monkeys^8^. The dorsolateral striatum is a primary target of midbrain dopamine neuron terminals and dopaminergic neurotransmission is important in habit formation^9^. Furthermore, the dorsolateral striatum receives excitatory glutamatergic input from cortical neurons and consistently the N-methyl-D-aspartic acid (NMDA) glutamate receptor plays an important role in procedural learning task performance^10^. Ultimately striatum-associated learning likely depends on the integration of dopamine and glutamate signals, which both are major contributors to striatal synaptic plasticity.

The striatum is the major input nucleus of the basal ganglia and is composed mainly of GABAergic projecting medium spiny neurons. Within the striatum, two forms of long-lasting synaptic plasticity have been described at glutamatergic cortico-striatal synapses, namely long-term depression (LTD) and long-term potentiation (LTP). By far, the most commonly reported form of cortico-striatal plasticity is LTD that can be induced in response to high-frequency stimulation (HFS) *in vitro*. The induction of striatal LTD requires postsynaptic depolarization and endocannabinoid release^11^,^12^. In contrast, striatal LTP studies have employed widely varied techniques and indeed it has been challenging to induce striatal LTP *in vitro*^11^,^13^,^14^,^15^,^16^,^17^,^18^. It has been reported that HFS can induce either LTD or LTP in cortico-striatal slices, depending on stimulating electrode placement, striatal subregion, and age of animal^19^,^20^. For example, dorsomedial striatum exhibited chiefly HFS-induced LTP. Dorsolateral striatum also exhibited HFS-induced LTP in young mice^19^. A recent study reported that theta burst stimulation effectively induces LTP in dorsomedial striatum, while having limited effects in the dorsolateral region^18^. Considering the entire body of evidence on striatal LTP and LTD, obvious discrepancies become apparent and further research is required to better understand these processes in dorsolateral striatum.

Striatal synaptic plasticity is depending on dopaminergic and glutamatergic neurotransmission. Consistently striatal LTP induction has been reported to be NMDAR- and dopamine-dependent^21^,^22^. It is thought that dopaminergic and glutamatergic neurotransmission trigger intracellular signaling cascades that contribute to the induction, expression and maintenance of striatal LTP^23^. For example, increases in intracellular calcium are required for striatal LTP expression^21^,^22^. At the level of intracellular signal transduction, the protein kinase, Cdk5 has been shown to modulate dopamine signaling in striatum through the regulation of the protein phosphatase-1 inhibitor, DARPP-32 and the cAMP phosphodiesterase, PDE4^24^,^25^. Cdk5 has also been implicated in the synthesis and release of dopamine^26^,^27^. Cdk5 is a proline-directed serine/threonine kinase that is activated through interaction with its cofactors p35 or p39^28^. This kinase has been implicated in numerous CNS processes, including cortical layer formation, neurotransmission, and mnemonic functions^29^,^30^. Cdk5 also modulates presynaptic neurotransmitter release and calcium entry through the phosphorylation of voltage gated calcium channels^31^,^32^. Recently it was demonstrated that Cdk5 modulates synaptic plasticity, learning, and memory through phosphorylation of the NMDA receptor (NMDAR) subunit, NR2B in hippocampus^33^,^34^. Here, we further characterize cortico-striatal synaptic plasticity in mice via extracellular field recordings, and assess the role of Cdk5 in striatal LTP and motor skill learning.

## Results

### Long-Term Plasticity in cortico-striatal slices

**Figure 1:**
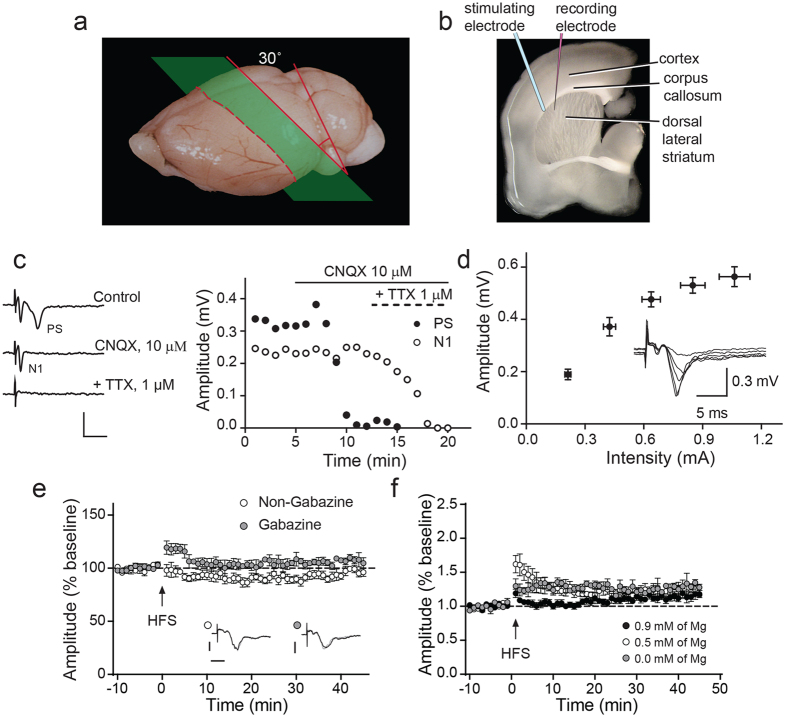
Characterization of a consistent recording paradigm for mouse cortico-striatal synaptic plasticity. **(a)** Schematic demonstrating the oblique coronal section used to derive slices for cortico-striatal field recordings. **(b)** Schematic showing stimulating and recording electrode placement in oblique coronal slice. **(c)** Example traces with N1 and PS deflections labeled (left). Time-course for N1 and PS components of fEPSP recordings under control conditions and in response to the application of CNQX and TTX are shown (right). **(d)** Input-output curve for the amplitude of the PS component of cortico-striatal fEPSP, example traces at different intensities are shown (inset). Data shown are mean ± s.e.m, *n* = 8. **(e)** Tetanic stimulation (HFS) in absence or presence of of gabazine (GABA_A_ antagonist). Data shown are mean ± s.e.m, *n* = 7. **(f)** Effect of magnesium concentration on fEPSP amplitude in presence of gabazine. Data shown are mean ± s.e.m, *n* = 7.

Most neurophysiological studies of the striatum commonly use coronal sections to record from. To optimize the integrity of this circuitry, oblique coronal sections were used for extracellular field recordings (Fig. 1a). These recordings were performed in the rostral, dorsal, and lateral portions of the striatum while stimulating in the corpus callosum (Fig. 1b). Square pulse current stimulations elicited field responses that exhibited two negative spikes, N1 and PS or population spike (Fig. 1c). The larger PS component was dependent upon glutamatergic synaptic transmission, as it was completely ablated by the competitive AMPA/kainate receptor antagonist, CNQX. In contrast, the relatively minor initial N1 deflection was unaffected by CNQX, similar to the presynaptic fiber volley observed in hippocampal field recordings. Furthermore, both PS and N1 deflections were abolished by addition of TTX, demonstrating both components required functional voltage-gated sodium channels. The PS deflection showed a typical stimulus-to-fEPSP amplitude ratio (input-output) for synaptic stimulation (Fig. 1d). These results suggested that the smaller initial N1 deflection is non-synaptic, while the PS deflection is synaptically driven^20^. Therefore, all evaluations of striatal plasticity in this study were subsequently conducted based on measurement of the amplitudes of PS deflections.

HFS of these oblique cortico-striatal slices in the presence of magnesium concentrations mimicking physiological conditions (1.3 mM) and in the absence of a GABA_A_ receptor antagonist produced a transient reduction in fEPSP amplitude, which returned to baseline after 40 min of recordings (0.32 ± 0.03 mV baseline compared to 0.31 ± 0.02 mV; *p* > 0.05; *n* = 7, paired *t*-test) (Fig. 1e). Also, no post-tetanic potentiation (PTP) was observed after HFS. Addition of the GABA_A_ antagonist, Gabazine (3 μM) resulted in a small PTP following HFS. However, no LTP was induced (0.26 ± 0.02 mV baseline compared to 0.28 ± 0.01 mV; *p* > 0.05; *n* = 7, paired *t*-test). To further optimize conditions for LTP induction, the effects of magnesium concentration in the presence of Gabazine were next explored (Fig. 1f). Reduction of magnesium from 1.3 to 0.9 mM resulted in a small increase in PTP and a latent induction of low level LTP (0.28 ± 0.03 mV baseline compared to 0.32 ± 0.02 mV; *p* > 0.05; *n* = 7, paired *t*-test). Reducing magnesium further, to 0 mM resulted in a more immediate and higher level of LTP (0.28 ± 0.02 mV baseline compared to 0.32 ± 0.06 mV; *p* > 0.05; *n* = 7, paired *t*-test). However, the complete absence of magnesium from the recording solution resulted in a destabilization of fEPSP response and greater variability between slice recordings, possibly due to aversive physiological conditions. Based on these empirical experiments, all subsequent recordings were taken in the presence of Gabazine and 0.5 mM magnesium. These results also demonstrate that blocking inhibitory GABA_A_ receptors (Gabazine) and potentiation of NMDAR function, by lowering magnesium, are essential to the induction of LTP in these preparations of dorsolateral striatum.

### Long-term potentiation in cortico-striatal circuitry of the dorsolateral striatum is facilitated by dopamine and dependent upon D1-dopamine receptor activation

**Figure 2:**
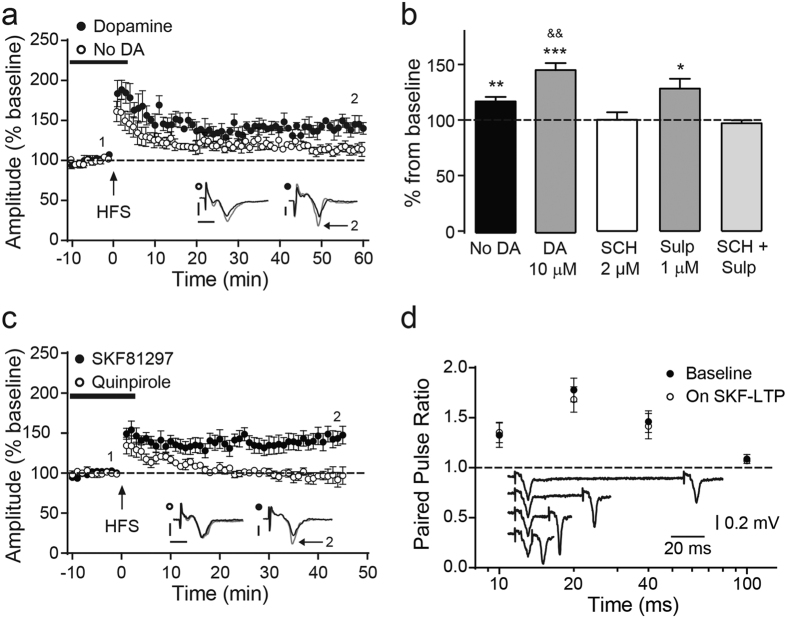
Dopamine modulates striatal synaptic plasticity. **(a)** Time-course of fEPSP amplitude plotted as percent of baseline (0–10 min) showing the effect of dopamine on HFS-induced LTP. Each point is derived from the mean. Representative traces shown as insets were taken at baseline just before HFS (black, 1) and at end of recordings (gray, 2). **(b)** Summary showing effects of dopamine, and D1- (SCH23390) and D2-like (sulpride) dopamine receptor antagonists on fEPSP amplitude, derived from final 5 min of time-course recording after HFS. **(c)** Time-course shows the effect of a D1-dopamine receptor agonist (SKF81297, 2 μM) or D2-dopamine receptors agonist (quinpirole, 1 μM) on LTP. **(d)** Paired pulse ratio at different intervals shows no changes during D1-induced LTP compared to the baseline, representative traces from baseline are shown as inset. Representative traces shown as insets were taken at baseline just before HFS (black) and at end of recordings (gray). All data shown are mean ± s.e.m., *p < 0.05, **p < 0.01, ***p < 0.001 vs. baseline; ^&&^*p* < 0.01 vs. No DA, *n* = 7, unpaired *t*-test. Scale bar; vertical = 0.2 mV, horizontal = 5 ms.

It is well established that dopamine neurotransmission contributes to striatal synaptic plasticity^21^,^22^. Here the effect of dopamine on striatal LTP was assessed. In the absence of dopamine, HFS of cortico-striatal circuitry induced transient post-tetanic potentiation (PTP) and a small but significant increase in the fEPSP amplitude (0.28 ± 0.02 mV baseline compared to 0.33 ± 0.02 mV 60 min after HFS; **p < 0.01; *n* = 7, paired *t*-test). The fEPSP amplitude increase was maintained for at least 60 min of recording (Fig. 2a). Incubation of cortico-striatal slices with dopamine (10 μM) for 15 min before HFS, while baseline recordings (−10 to 0 min) were taken and perfusion was halted 3 min following HFS. Dopamine perfusion did not change the fEPSP amplitude during baseline stimulation. However, dopamine perfusion significantly increased post-HFS fEPSP amplitude (0.27 ± 0.02 mV baseline compared to 0.40 ± 0.04 mV; **p < 0.01; *n* = 7, paired *t*-test) compared to untreated control slices. Furthermore, dopamine perfusion before and during the HFS significantly increased LTP (114 ± 2.6% without dopamine compared to 146 ± 6.3% with dopamine, ***p < 0.001, *n* = 7, unpaired *t*-test) (Fig. 2b).

To explore the action of endogenous dopamine release on cortico-striatal plasticity, the effect of dopamine receptor-selective antagonists on plasticity was assessed (Fig. 2b). The potentiation of fEPSP amplitude induced by HFS was completely abolished in presence of the D1-dopamine receptor antagonist, SCH23390 (0.36 ± 0.04 mV baseline compared to 0.37 ± 0.06 mV; *p* > 0.05, *n* = 5, paired *t*-test). In contrast, the D2-dopamine receptor antagonist, sulpiride, did not impaire LTP induction (0.31 ± 0.02 mV baseline compared to 0.38 ± 0.05; *p < 0.05; *n* = 6, paired *t*-test). Combined treatment with both SCH23390 and sulpiride again ablated LTP induction (0.28 ± 0.02 baseline to 0.26 ± 0.02; *p* > 0.05, *n* = 5, paired *t*-test). These results suggest that the relatively moderate potentiation in fEPSP amplitude and LTP induction that occurs in absence of exogenous dopamine perfusion is dependent upon endogenous dopamine release and D1-dopamine receptor activation.

To further explore the role of dopamine signaling in cortico-striatal plasticity, selective agonists of D1- and D2-dopamine receptors were used (Fig. 2c). The ability of dopamine to facilitate LTP (see Fig. 2a) was replicated by addition of the D1-dopamine receptor agonist, SKF81297 (2 μM), which potentiated fEPSP amplitude (0.29 ± 0.03 mV baseline compared to 0.42 ± 0.06 mV; *p < 0.05, *n* = 8, paired *t*-test). In contrast, treatment of slices with the D2-dopamine receptor agonist, quinpirole prior to and during HFS did not facilitated LTP, but instead, prevented LTP induction in comparison to that induced by HFS in untreated slices (0.38 ± 0.04 mV baseline to 0.36 ± 0.05 mV; *p* > 0.05, *n* = 6, paired *t*-test). To determine whether striatal LTP was modulated by pre- or postsynaptic mechanism, paired pulse facilitation paradigm during baseline (before SKF) and 45 min after HFS during SKF-induced LTP was assessed. These results show that sustained LTP is modulated by postsynaptic changes (Fig. 2d). Taken together, these data demonstrate that cortico-striatal plasticity is dopamine-dependent, and that the actions of dopamine that are critical to LTP induction are mediated via D1-dopamine receptor activation. Furthermore, D2-dopamine receptor signaling may oppose LTP expression in the dorsolateral striatum.

### Striatal LTP is mediated by NMDA receptors and CaMKII

NMDAR function has been well characterized in striatal plasticity^35^. To further understand the neurophysiological basis of the integration of NMDAR- and Dopamine receptor neurotransmission, the contribution of NMDAR function and its downstream signaling in dopamine-facilitated cortico-striatal potentiation was examined.

**Figure 3:**
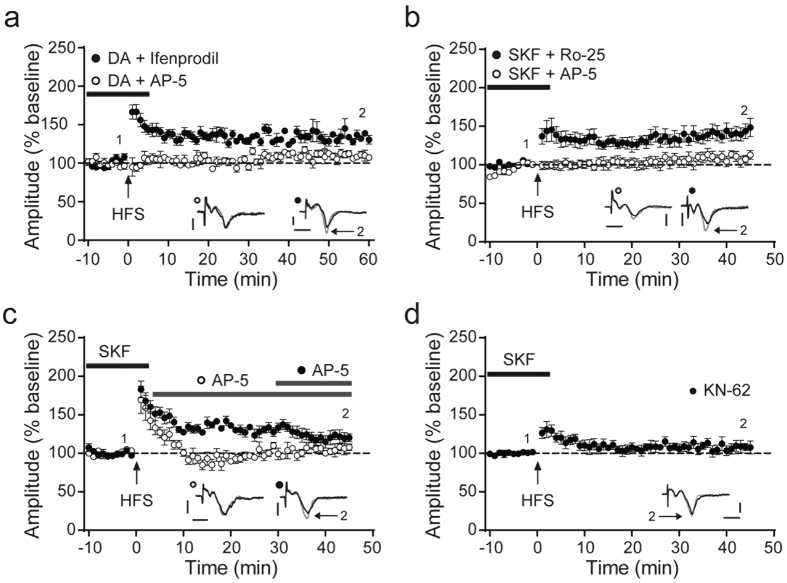
Dopamine receptor-facilitated cortico-striatal LTP is mediated by NMDA receptors and CaMKII. **(a)** Dopamine-facilitated LTP induced in the presence of either the general NMDA receptor antagonist AP-5 or the NR2B-selective receptor antagonist, ifenprodil (3 μM) is shown with representative traces. **(b)** Effect of AP-5 versus the NR2B-selective receptor antagonist, Ro-256981 (3 μM) on D1-dopamine receptor agonist-facilitated LTP is shown with representative traces. **(c)** Time-course of the fEPSP amplitude showing the temporal effect of NMDA receptor block by AP-5 addition 3 or 30 min after D1-dopamine receptor activation and HFS. **(d)** Effect of CaMKII inhibitor (KN-62) on D1-dopamine receptor facilitated LTP. All data represent means ± s.e.m., *n* = 6–8. Representative traces shown as insets were taken at baseline (black) just before HFS and at end of recordings (gray), scale bar; vertical = 0.2 mV, horizontal = 5 ms.

Treatment with the NMDAR antagonist, AP-5, impaired dopamine-facilitated LTP induction (0.21 ± 0.03 mV baseline to 0.25 ± 0.05 mV; *p* > 0.05; *n* = 8, paired *t*-test) (Fig. 3a) and completely blocked post-tetanic potentiation (PTP). Moreover, AP-5 prevented PTP and LTP in the presence of the D1R-selective agonist SKF81297 (Fig. 3b), indicating that D1-dopamine receptor agonist-enhanced plasticity was NMDAR-dependent. In contrast, the NR2B selective antagonist, ifenprodil (3** **μM) did not affect LTP induction. Consistently, the NR2B-selective antagonist, Ro 25-6981 (Ro-25 3** **μM) had no effect on D1 agonist-facilitated LTP (0.34 ± 0.04 mV baseline compared to 0.48 ± 0.07; *p < 0.05, *n* = 5, paired *t*-test). These results indicate that NR2B-mediated NMDA function does not contribute appreciably to the overall NMDAR-dependence of cortico-striatal plasticity.

In the above experiments, blocking NMDAR prior to and during HFS prevented PTP and LTP induction. To understand better the temporal contribution of NMDAR to cortico-striatal plasticity, AP-5 was added at different time points following HFS in the presence of SKF81297 (Fig. 3c). Addition of AP-5 three min after HFS resulted in decline in fEPSP amplitude completely to baseline levels following PTP (0.32 ± 0.02 mV baseline compared to 0.29 ± 0.03; *p* > 0.05, *n* = 6, paired *t*-test). In contrast, addition of AP-5 after a post-HFS delay period of 30 min resulted in an apparent partial attenuation of LTP (0.28 ± 0.01 mV baseline to 0.34 ± 0.02 mV; *p < 0.05, *n* = 7, paired *t*-test), despite the expected induction of PTP and response amplitude potentiation prior to AP-5 perfusion. Together these data underline the critical contribution of NMDAR function to the induction and maintenance of the elevated response state that characterizes LTP.

NMDAR are thought to activate downstream calcium-dependent signaling events that mediate plasticity and learning such as the activation of the protein kinase, CaMKII^36^. Thus we assessed the effect of the selective CaMKII inhibitor KN-62 (10** **μM) on cortico-striatal plasticity. Inhibition of CaMKII by pre-incubation of slices with KN-62 (1 h) effectively inhibited the potentiation induced by HFS in the presence of SKF81297 (0.29 ± 0.03 mV baseline compared to 0.31 ± 0.04 mV; *p* > 0.05, *n* = 8, paired *t*-test) (Fig. 3d). This result is consistent with the activation of CaMKII, likely downstream of NMDAR activation, as an important contributor to cortico-striatal plasticity.

### Inhibition of Cdk5 impairs dorsolateral striatal synaptic plasticity

**Figure 4:**
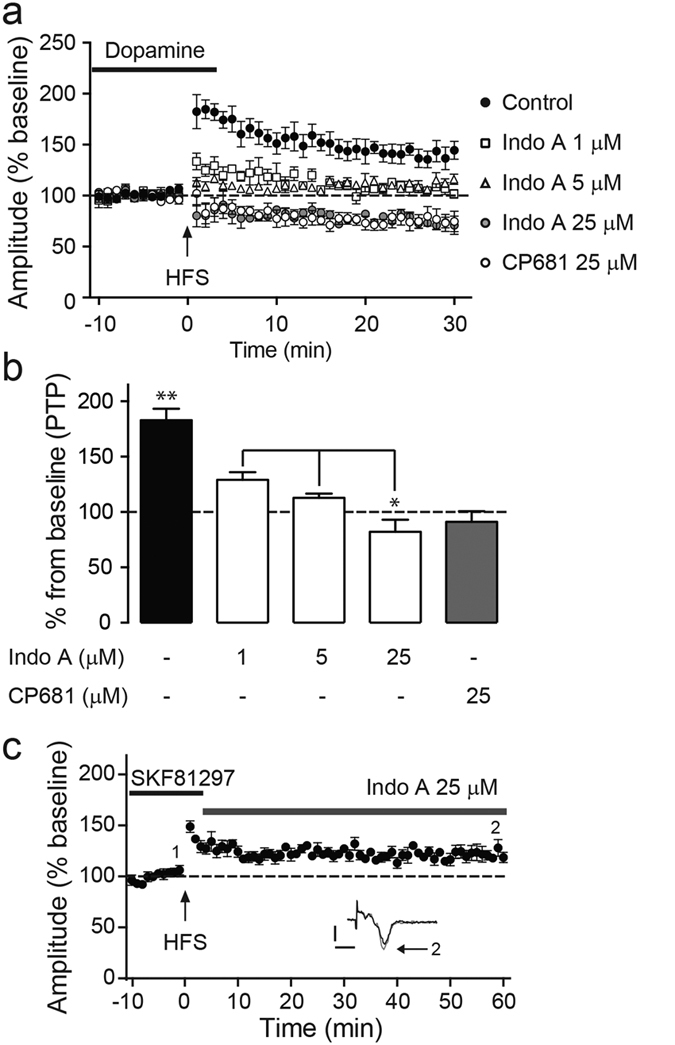
Cdk5 inhibition impairs dopamine-facilitated LTP induction in cortico-striatal circuitry. **(a)** Time course showing effect of the indicated treatment with Cdk5 inhibitors on dopamine-facilitated increases in PTP and fEPSP amplitude following HFS. **(b)** Summary of the dose-dependent effects of Cdk5 inhibitors on PTP. Bars represent mean of the fEPSP percent over the first 3 min after HFS. **(c)** Effect of post-HFS addition of the Cdk5 inhibitor, Indo A on sustained D1-facilitated LTP. All data shown are means ± s.e.m., *p < 0.05, **p < 0.01; *n* = 6, unpaired and paired *t*-test.

The neuronal protein kinase Cdk5 has been implicated as a downstream effector of NMDAR function and dopamine signaling^24^,^34^. Moreover, loss of Cdk5 increases neuronal excitability in striatal neurons^37^. To gain a better understanding of the role of Cdk5 in striatal plasticity, the effect of Cdk5 inhibition on dopamine-facilitated synaptic potentiation was examined. Field recordings from slices pre-incubated with selective and potent Cdk5 inhibitors, either Indolinone A (Indo A) or CP681301 (CP681), for 1 h did not affect input-output curves, indicating no significant changes on the basal synaptic cortico-striatal circuitry (data not shown). However, Cdk5 inhibition attenuated dopamine-facilitated LTP and PTP (Fig. 4a,b) in a concentration-dependent manner. Interestingly, slices treated with 25 μM IndoA or CP681 depressed fEPSP amplitudes to lower than baseline levels (IndoA: 100 ± 01% baseline compared to 73 ± 2%; or CP681: 99 ± 2% baseline compared to 76 ± 8%; *p < 0.05, *n* = 5, paired *t*-test). Importantly, LTP maintenance was not affected when the slices were treated with IndoA three minutes after HFS (0.32 ± 0.02 mV baseline compared to 0.38 ± 0.02 mV; *p < 0.05, *n* = 7, paired *t*-test) (Fig. 4c). These results indicate that Cdk5 activity is important for the induction of dopamine-facilitated LTP in cortico-striatal circuitry, but not for the maintenance of LTP.

### Brain-wide conditional knockout of Cdk5 in adult mice impairs dorsolateral striatal synaptic plasticity

**Figure 5:**
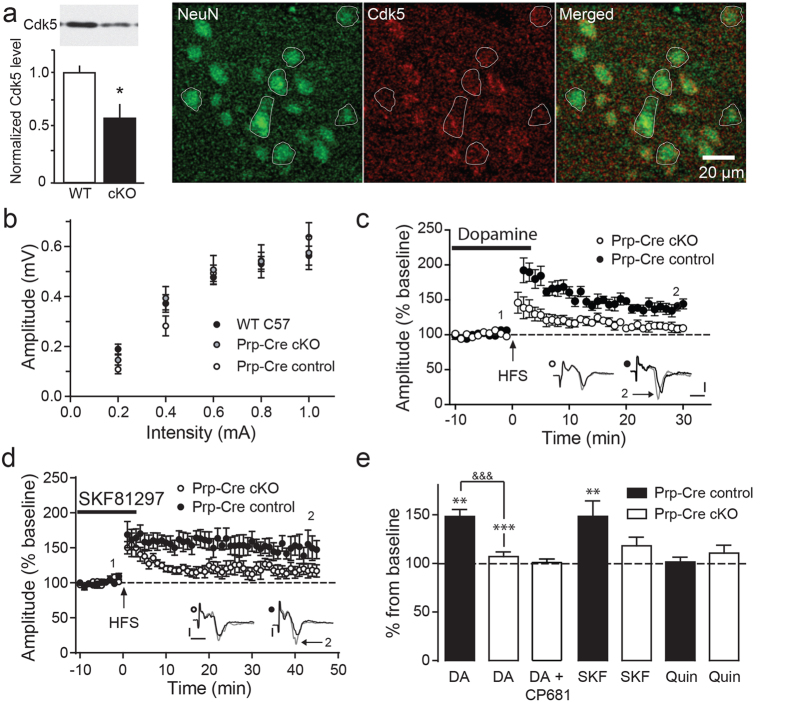
Conditional knockout of Cdk5 impairs cortico-striatal synaptic plasticity. **(a)** Quantitative immunoblot analysis and immunostaining showing Cdk5 cKO in dorsolateral striatum. Immunoblot of dorsal striatal lysate for Cdk5 with quantitation is shown (left). Immunostain is shown with medium spiny neurons positive for neuronal nuclear marker NeuN (green), but negative for Cdk5 (red) outlined. Data represent means ± s.e.m., *p* < 0.05, *t*-test, *n* = 4. **(b)** Prp-ER^T^Cre-mediated brain-wide Cdk5 cKO in adult mice had no significant effect on input-output compared to liter mate controls or WT mice. **(c)** Time-course showing the effect of Cdk5 cKO on dopamine-facilitated cortico-striatal LTP. The representative traces were taken at the time points indicated. **(d)** Time-course showing the effect of the D1-dopamine receptor selective agonist, SKF81297, on LTP in control vs. Cdk5 cKO mice, **(e)** Summary of effects of dopamine, SKF81297, the selective D2-dopamine receptor agonist, quinpirole, and the Cdk5 inhibitor CP681 in control vs. Cdk5 cKO mice under the treatment conditions indicated. All data shown are means ± s.e.m., *p < 0.05, **p < 0.01, ***p < 0.001 vs. baseline; paired *t*-test, ^&&&^*p* < 0.001; unpaired *t*-test, *n* = 6–8. Representative traces shown as insets were taken at baseline (black) just before HFS and at end of recordings (gray), scale bar; vertical = 0.2 mV, horizontal = 5 ms.

In addition to pharmacological inhibition, conditional knockout (cKO) transgenics provide an effective complementary approach for understanding the role of Cdk5 in striatal synaptic plasticity. Thus, we utilized Cdk5 cKO mice where Prp-ER^T^-Cre mediated the loss of Cdk5 throughout the brain of adult mice in response to 6-hydroxytamoxifen treatment (Prp-Cre cKO)^33^. These mice exhibit approximately 50% of the level of dorsal striatal Cdk5 that occurs in littermate controls (Fig. 5a). Loss of Cdk5 staining was evident in almost half of all dorsal striatal medium spiny neurons. Cdk5 cKO had no effect on input-output curves in comparison to either littermate controls or age-matched WT mice (Fig. 5b), indicating that Cdk5 loss had no general effect on cortico-striatal synaptic connectivity.

Consistent with the results of pharmacological Cdk5 inhibition, dopamine-facilitated synaptic potentiation was significantly impaired in slices from Cdk5 cKO mice (0.41 ± 0.05 mV baseline compared to 0.43 ± 0.04 mV; *p* > 0.05, *n* = 6, paired *t*-test) (Fig. 5c,e). Slices from Cdk5 cKO mice exhibit a significant reduction in the amplitude from 148 ± 7% in control mice to 105 ± 4% (Fig. 5c). To evaluate the level of Cdk5 knockout and its functional effects, cortico-striatal slices from Cdk5 cKO mice were treated with the Cdk5 inhibitor CP681 (25 μM) and dopamine-facilitated LTP was assessed (see summary data in Fig. 5e). Cdk5 inhibition did not affect dopamine-facilitated synaptic potentiation in Cdk5 cKO, demonstrating effective loss of Cdk5, as well as complementarity between pharmacological and genetic inhibition of Cdk5.

To study the relative contributions of D1- versus D2-dopamine receptors to the facilitation of cortico-striatal plasticity in Cdk5 cKO, the effect of D1-dopamine receptor selective agonist SKF81297 and D2-dopamine receptor selective agonist quinpirole on fEPSP amplitude and LTP was assessed in slices from Cdk5 cKO and controls. In line with the results obtained with dopamine treatment in Cdk5 cKO (Fig. 5c), the potentiating effect of SKF81297 was abolished in Cdk5 cKO mouse slices (0.31 ± 0.04 mV baseline compared to 0.36 ± 0.07 mV; *p* > 0.05, *n* = 6, paired *t*-test). Slices from Cdk5 cKO mice exhibit a significant reduction in the amplitude from 149 ± 16% compared to 116 ± 10%. In contrast, the D2-receptor selective agonist quinpirole had no effect on HFS-induced fEPSP potentiation either in control slices (0.35 ± 0.02 mV baseline to 0.36 ± 0.02 mV; *p* > 0.05, *n* = 6, paired *t*-test) or Cdk5 KO slices (0.35 ± 0.02 mV baseline compared to 0.39 ± 0.04 mV; *p* > 0.05, *n* = 5, paired *t*-test). These data confirm that the effect of dopamine on cortico-striatal LTP is D1-dopamine receptor-mediated and that Cdk5 activity contributes critically to this plasticity.

### Dorsolateral striatum-specific viral-mediated Cdk5 KO attenuates striatal plasticity, alters locomotor behavior, and impairs motor learning

Cdk5 has been implicated in addiction, locomotor and stress-induced behavior^25^,^37^. To better understand the role of Cdk5 in dorsolateral function, the effect of dorsolateral specific Cdk5 loss on neurophysiology and behavior was tested. For this purpose a viral strategy was used to induce Cdk5 loss specifically in dorsolateral striatum. Mice homozygous for the floxed Cdk5 allele were bilaterally infused into dorsolateral striatum with rAAV2 vector expressing a Cre-GFP fusion. The control group was homozygous floxed Cdk5 mice infused with PBS and WT littermates infused with rAAV-Cre-GFP vector.

**Figure 6:**
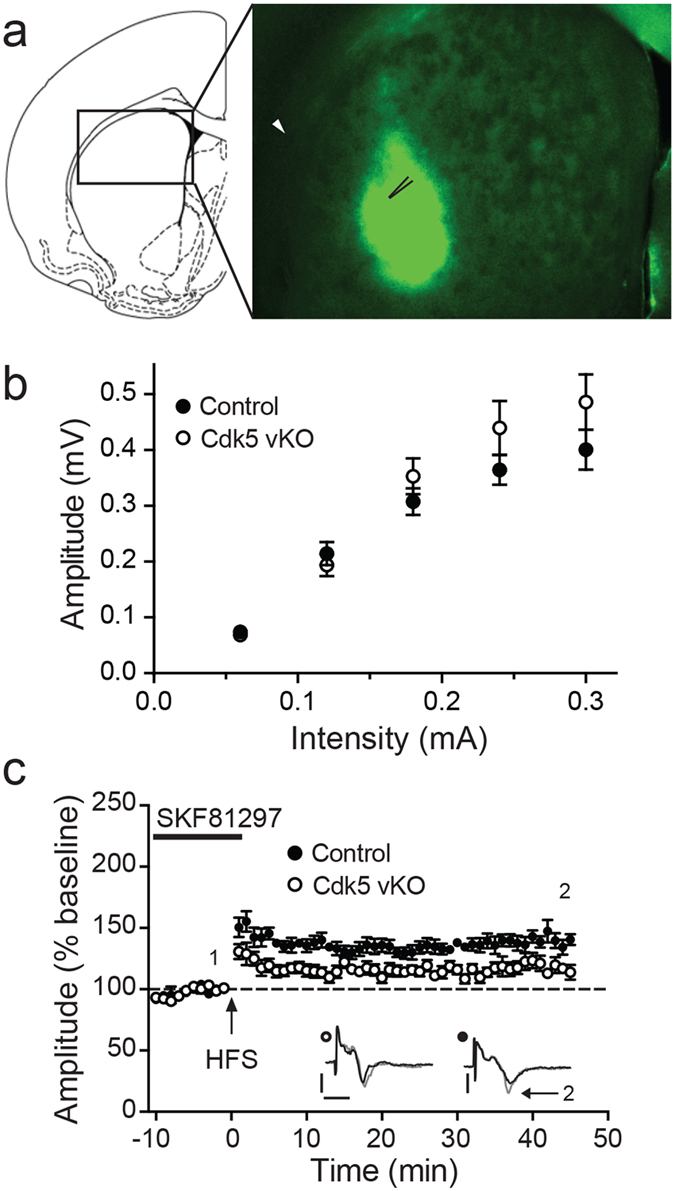
Dorsolateral striatal-specific viral-mediated Cdk5 KO impairs cortico-striatal LTP. **(a)** Epifluorescent detection of viral mediated Cre-GFP expression in oblique coronal section used for fEPSP recordings. Placement of the recording pipette in the fluorescent area (unfilled arrowhead) and the stimulation electrode (white arrowhead) in the corpus callosum is indicated with diagram of coronal section to demonstrate anatomical position. **(b)** The input-output curves for dorsolateral striatal-specific viral-mediated Cdk5 KO (Cdk5 vKO) and control are shown. **(c)** Effect of Cdk5 vKO on D1-dopamine receptors agonist-facilitated LTP. All data are means ± s.e.m.; *n* = 8. Representative traces shown as insets were taken at baseline (black) just before HFS and at end of recordings (gray), scale bar; vertical = 0.2 mV, horizontal = 5 ms.

**Figure 7:**
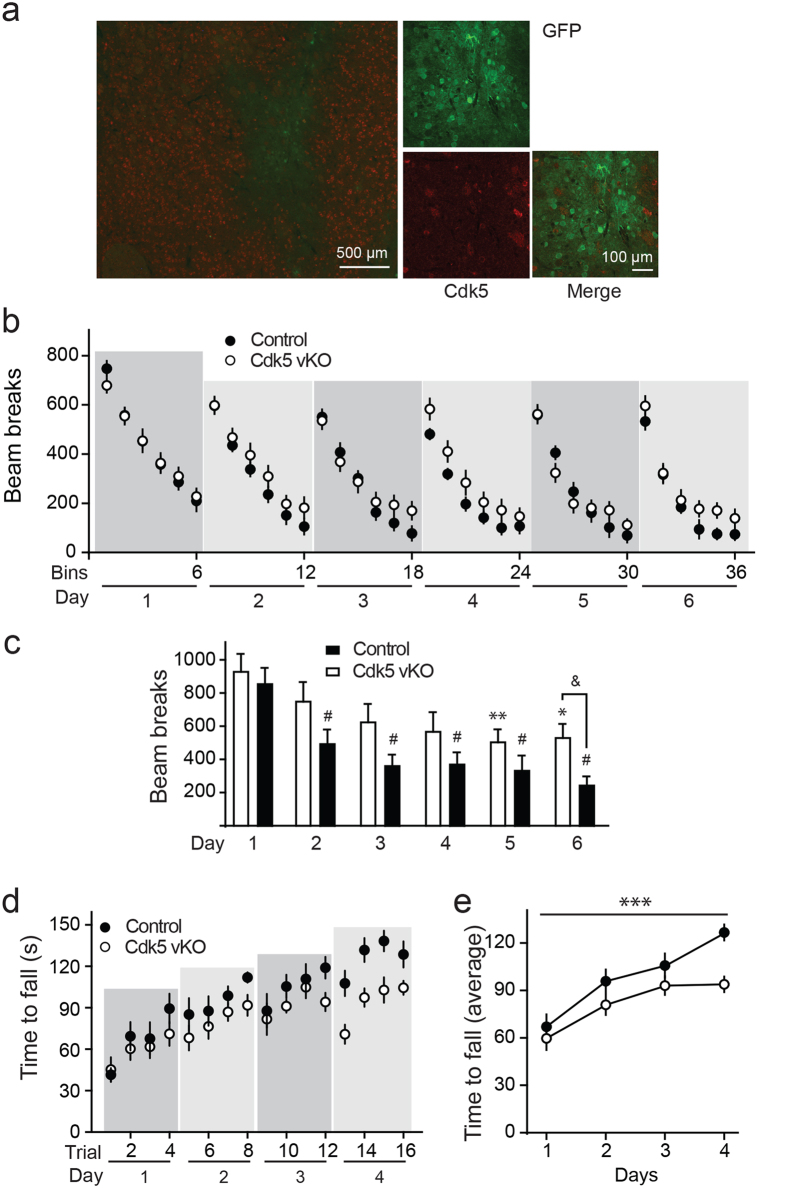
Dorsolateral striatal-specific Cdk5 vKO alters locomotor behavior and motor learning. **(a)** Immunostain showing viral mediated Cre-GFP expression as a circumscribed area at the injection site within dorsolateral striatum, which stains positive for GFP (green) with corresponding loss of Cdk5 (red). **(b**,**c)** Effect of dorsolateral striatal-specific Cdk5 vKO on locomotor activity. Locomotor activity (bream breaks) in 20 min bins for 2 h daily sessions for 6 days is shown. The shaded panels behind the data points denote the different test days (**b**). Bar graph of average locomotor activity for 6 days is shown (One-way ANOVA with multiple comparisons correction for Control: ^#^p = 0.0001, compared with day1, for Cdk5 vKO; *p < 0.05, **p < 0.01 compared with day1, n = 13–15. Multiple t tests of Two-way RM ANOVA by group: ^&^p = 0.0489, n = 13–15) (**c**). **(d**,**e)** Effect of Cdk5 vKO on rotarod performance. Time spent on the rotating rod for each trial, 4 trials each day, for 4 days is shown. The shaded panels behind the data points denote the different test days (**d**). Average time spent on rod for day 1–4 (two-way RM ANOVA: interaction *F*_(3, 51)_ = 2.974, *p* = 0.04; group *F*_(1, 17)_ = 5.03, *p* = 0.038; day *F*_(3, 51)_ = 39.81, *p* < 0.0001, *n* = 9–10) (**e**). All data represent mean ± s.e.m.

To evaluate the effect of dorsolateral striatal-specific viral-mediated Cdk5 KO (Cdk5 vKO) on the neurophysiology of cortico-striatal circuitry, field recordings were used to explore D1-dopamine receptor-facilitated synaptic plasticity. For these experiments, the recording electrode was placed in the GFP-expressing regions of slices from Cdk5 vKO mice that were visualized by epifluorescence, with the stimulating electrode located in corpus callosum (Fig. 6a). The GFP-expressing region was confirmed to be Cdk5 KO by immunohistochemistry (see Fig. 7a). Input-output analysis showed no significant effect on peak amplitudes obtained at maximal stimulation, suggesting no significant change in the basal cortico-striatal network in the Cdk5 vKO (Fig. 6b). However, the potentiating effect of SKF81297 was attenuated in Cdk5 vKO mouse slices (0.28 ± 0.02 mV baseline compared to 0.33 ± 0.03 mV; **p < 0.01, *n* = 8, paired *t*-test). As was observed with pharmacological as well as brain-wide cKO, LTP was significantly impaired by dorsolateral striatal-specific Cdk5 vKO compared to control mice (140 ± 5% for control vs. 117 ± 4% for Cdk5 vKO; **p < 0.01, *n* = 8, unpaired *t*-test). Furthermore Cdk5 vKO exhibited an apparent reduction in PTP. Both control and vKO mice exhibited LTP, which was stably maintained after 45 min of HFS (Fig. 6c). This data shows that Cdk5 within dorsolateral striatum affects D1-dopamine receptor function and thus SKF-facilitated synaptic plasticity possibly via the modulation of dopamine signaling supporting our previous results.

Motor performance is dependent upon neurotransmission in the dorsal striatum^38^ and cortico-striatal circuitry contributes to aspects of motor learning^39^,^40^. To understand how striatal Cdk5 contributes to these functions, behavioral testing was conducted with Cdk5 vKO and control mice 3 weeks after intra-striatal Cre-GFP AAV2 infusion. Viral gene transfer-mediated dorsolateral-specific KO was confirmed by immunostaining for Cdk5 and GFP (Fig. 7a).The GFP-expressing region colocalized with areas lacking Cdk5, demonstrating dorsolateral-specific viral-mediated Cdk5 KO. To assess the effects of dorsolateral striatal-specific Cdk5 vKO on motor performance, locomotor activity was examined in Cdk5 vKO and controls for 2 h each day over 6 consecutive days (Fig. 7b,c). In this paradigm, both Cdk5 vKO and control mice demonstrated typical transfer arousal and exploratory behavior. Locomotor activity for both groups was comparable on the first day of testing indicating that loss of Cdk5 does not generally induce hyperactivity. The locomotor performance of both groups decreased over the daily sessions during days 2–6, consistent with the process of environmental habituation. Interestingly, Cdk5 vKO exhibit attenuated habituation during the 2 h sessions that becomes more apparent over the course of 6 days. (Fig. 7b,c; one-way ANOVA with multiple comparisons correction: Control, ^#^*p* = 0.0001, compared with day1, *n* = 13; Cdk5 vKO; *p < 0.05, **p < 0.01 compared with day1, *n* = 15. Multiple *t*-test of two-way RM ANOVA by group: ^&^*p* = 0.0489, *n* = 13–15). Thus dorsolateral-specific Cdk5 vKO was implicated in locomotor behavioral changes and alteration in environmental habituation.

To assess the effect of dorsolateral striatal-specific Cdk5 loss in motor-coordination, -control, and -learning, rotarod performance was evaluated in Cdk5 vKO mice (Fig. 7d,e). The control mice exhibited improved performance within each day as well as over successive testing days indicating that motor learning occurred. In contrast, Cdk5 vKO mice showed significant deficit in rotarod performance on the fourth day (Fig. 7d; two-way RM ANOVA, interaction *F*_(3, 51)_ = 2.974, *p* = 0.04, *n* = 9–10). These results implicate Cdk5 in motor-coordination and -learning and support previous findings that dorsolateral striatum contributes to motor functions.

## Discussion

Better understanding of striatal function has been challenging due to subregion-, age-, and approach-specific discrepancies in the evaluation of striatal synaptic plasticity. Similarly, defining the role of Cdk5 in striatal function has been hindered by the congenital defects of constitutive knockouts^41^ and the lack of specificity of Cdk5 inhibitors (e.g. the Cdk5 inhibitor roscovitine may indirectly affect both PKA and calcium signaling^42^,^43^). Here, we have developed and characterized an approach to investigate plasticity in mouse cortico-striatal circuitry and interrogate the relative contributions of neurotransmitter receptors and intracellular signaling components. As part of this integrative approach we employed more selective inhibitors, different knockout strategies, and assessment of relevant striatal-mediated behaviors in mice to better understand striatal plasticity and the role of Cdk5. Our findings indicate that Cdk5 contributes in dopamine-facilitated dorsolateral cortico-striatal synaptic plasticity and motor-associated behavior.

Initial studies, conducted mostly in rats, predominantly emphasized LTD plasticity in cortico-striatal circuitry^13^,^44^,^45^. However subsequent studies demonstrated LTP in other preparations, such as *in vivo* recordings under anesthesia^14^,^46^,^47^. *In vivo* recordings also showed LTD, which could be prevented or reversed by concomitant stimulation of substantia nigra^48^. Alternate approaches to studying LTP include the use of sharp electrodes, perforated patch, spike-timing dependent plasticity, or extracellular recordings under specific conditions such as magnesium-free solution^11^,^14^,^16^,^17^,^20^. Also, recent studies shows reliable LTP induction in dorsomedial striatum by theta burst stimulation^18^,^49^. To better understand the physiological conditions under which LTP occurs in cortico-striatum, we first surveyed dorsolateral field responses to tetanic stimulation of corpus callosum in coronal oblique mouse brain sections. This allowed isolation of an N1 spike, which is fiber volley-related, and a synaptically driven PS component, as has been previously described in rat preparations^20^.

Isolating the PS, we demonstrated that LTP was consistently induced in mouse striatum by tetanic stimulation in the presence of a GABA_A_ antagonist. This is consistent with previous reports showing the role of GABA_A_ transmission in LTP induction^50^,^51^. Here, cortico-striatal LTP was dependent upon and facilitated by D1-dopamine receptor activation, and also required the activation of NMDAR, in agreement with previous reports^11^,^17^,^52^,^53^. Consistent with the present study, LTP induction in this circuitry in rats is impaired by dopamine terminal denervation^54^,^55^.

While dopamine markedly enhanced cortico-striatal LTP, HFS of corpus callosum also likely induces localized endogenous dopamine-release in dorsal striatum^56^. This may explain how a small level of LTP induction can occur without addition of dopamine to the recording solution, while supporting dopamine-dependence for robust LTP induction in this circuitry. Here we demonstrated that the positive effects of dopamine upon cortico-striatal LTP was attributable to the activation of D1-dopamine receptors, in agreement with previous reports^11^,^17^,^52^,^53^. In sharp contrast, the activation of D2-dopamine receptors had no facilitating effect on LTP. Indeed blocking D2-dopamine receptors enhanced cortico-striatal LTP, likely due to inactivation of Gi-coupled D2-dopamine auto-receptors on presynaptic terminals.

Here, field recordings were used to explore cortico-striatal LTP, and thus the effects observed are not specific to direct (D1-dopamine receptor expressing) or indirect (D2-dopamine receptor expressing) pathway neurons. Studies of striatal plasticity at cellular resolution, using bacterial artificial chromosome (BAC) transgenics to tag cell type-specific neurons, support that LTP requires the activation of D1-dopamine receptors in MSN expressing D1-dopamine receptors. In contrast LTP induction in D2-dopamine receptor positive MSN was dependent on A2A-adenosine receptor activation^17^,^57^. Most of the studies describing LTP by intracellular recordings were performed using sharp electrodes or whole-cell recordings pairing strong and sustained depolarization with HFS^21^,^22^,^35^,^58^,^59^. Future studies validating and expanding these findings will investigate these mechanisms at the circuitry- and cell type-specific level.

In agreement with other studies, cortico-striatal dopamine-facilitated and D1-dopamine receptor-facilitated LTP was dependent upon NMDAR activation. A general NMDAR antagonist blocked LTP, while a selective antagonist of NR2B-containing NMDAR had no effect on LTP (Fig. 3), suggesting a limited role for receptors containing the NR2B subunit. NMDAR activation during and immediately after tetanic stimulation was essential to post-tetanic potentiation and LTP induction. However the sustained increased response that characterizes LTP was only partially NMDA-dependent, suggesting that NMDAR function is more critical during initiation rather than maintenance of LTP. In addition, LTP was blocked by CaMKII inhibition, suggesting calcium signaling downstream of NMDAR is critical to cortico-striatal plasticity.

To better understand the molecular mechanism underlying striatal plasticity, we explored the role of Cdk5 in cortico-striatal LTP. Here, attenuation of Cdk5 by pharmacological or transgenic means either reduced dopamine-facilitated LTP, or even resulted in LTD without affecting cortico-striatal synaptic connectivity. Cdk5 is a constitutively active protein kinase, which maintains the basal or homeostatic phosphorylation state of many proteins in both pre- and post-synaptic compartments. Previous studies uncovered an intricate interplay with multiple signaling pathways, including cAMP/PKA, GSK3 and CaMKII^24^,^25^,^60^,^61^. Cdk5 controls striatal cAMP/PKA signaling by phosphorylating and thereby facilitating activation of the various isoforms of the phosphodiesterase PDE4^25^. Consistently Cdk5-dependent phosphorylation of the protein phosphatase-1 inhibitor, DARPP-32 also inhibits PKA activity^24^. In striatum increases in intracellular cAMP and PKA activity are mainly driven by Gs-coupled D1 dopamine receptor activation. Therefore, it is possible that loss of Cdk5 could impair striatal plasticity by altering cAMP/PKA homeostasis. Moreover, Cdk5 regulates numerous additional mechanisms that are central for striatal plasticity and by which Cdk5 loss may contribute to the LTP impairment. For example, Cdk5 inhibition has been shown to impair striatal dopamine release, as well as dopamine biosynthesis^26^,^27^. Cdk5 inhibition may also impair synaptic vesicle cycling^62^, thereby affecting both dopamine and excitatory glutamate release.

Furthermore, Cdk5 as well as PKA are known to modulate NMDAR functions. Many studies address their role on NMDAR in hippocampal circuitry. Cdk5-dependent phosphorylation of the NMDAR subunit NR2A at Ser1232 is important for NMDAR conductance, and Cdk5 inhibition has been suggested to attenuate LTP induction via this mechanism^63^. Phosphorylation of NR2B at Ser1116 by Cdk5 controls NMDA-receptor trafficking and Cdk5 inhibition was found to facilitates hippocampal synaptic transmission and enhance memory formation^33^,^34^. NR2B is also phosphorylated by PKA at Ser1166 which increases NMDAR function^64^. Cdk5 may also regulate NMDAR function through its phosphorylation of PSD-95^65^. Moreover, activation of CaMKII via NMDA-dependent intracellular calcium promotes association between the Cdk5 activator, p35, and CaMKII^61^, suggesting a possible role for Cdk5-CaMKII interactions within the postsynaptic density in hippocampus.

The above stated mechanisms have been well characterized in hippocampus, but not in striatum. Hence, it is conceivable that these two brain regions use different molecular mechanisms for the expression of synaptic plasticity. For example, here we show that NR2B function is not essential to induce striatal plasticity, but plays an important role in hippocampus. Moreover, expression levels of DARPP-32 are very low in hippocampus and high in striatum. Clearly, additional mechanistic information will be needed in order to better understand how Cdk5 contributes to striatal synaptic plasticity.

In the current study, mice with dorsolateral striatal-specific Cdk5 loss exhibited altered locomotor behavior and motor learning deficits. The dorsolateral sensorimotor striatum (homologous to the putamen in primates) receives inputs from the sensorimotor cortex and is critical for procedural or implicit learning. This type of learning is required for the development of automatized responses that resemble habits^1^,^2^,^3^,^4^,^5^,^39^. Chemical lesion and dopamine denervation in the dorsolateral striatum disrupt habit formation in instrumental learning^9^,^66^. Furthermore, infusion of a NMDAR antagonist into dorsal striatum impairs motor skill performance^10^. Hence it is conceivable that future studies may unravel the functional interactions between striatal plasticity and motor functions.

## Methods

### Slice preparation

Adult male mice (12–16 weeks-old) were housed under 12 h light/dark cycle with food and water *ad libitum*. All procedures were conducted with approval and in accordance with the UT Southwestern Institutional Animal Care and Use Committee (IACUC) and NIH guidelines. Mice were anaesthetized by isoflurane and perfused transcardially with sucrose saline solution containing the following (in mM): 87 NaCl, 75 Sucrose, 2.5 KCl, 1.25 NaH_2_PO_4_, 7 MgCl_2_, 0.5 CaCl_2_, 25 NaHCO_3_, and 10 glucose (pH 7.4; saturated with 95% CO_2_, 5% O_2_). After decapitation, brains were removed and placed in ice-cold sucrose saline solution (4 °C). Oblique cortico-striatal slices (300 μm thick) were cut in 4 °C sucrose saline using a vibratome 3000 (Ted Pella, Redding, CA). Slices were transferred in saline solution containing the following (in mM): 125 NaCl, 2.5 KCl, 1.25 NaH_2_PO_4_, 1.3 MgCl_2_, 2 CaCl_2_, 25 NaHCO_3_, and 25 glucose (pH 7.4; saturated with 95% CO_2_; 5% O_2_). Slices were incubated for 20 min at 30 °C, then transferred to room temperature (22–25 °C) and allowed 1 h recovery.

### Electrophysiology

Slices were transferred to a recording chamber housed within an upright microscope stage (BX51WI Olympus America Inc., Pennsylvania, USA). Slices were visualized by infrared differential interference microscopy and a CCD Super Low Luminance camera (KT&C, Co., Ltd) adapted to the system. Slices were perfused continuously with oxygenated saline solution (2–3 ml/min).

#### Extracellular recordings

Field excitatory postsynaptic potential (fEPSP) were obtained in the presence of the GABA_A_ antagonist (SR95531, Gabazine, 2 μM) and were evoked by square current pulses (0.2 ms) at 0.033 Hz with a bipolar stimulation electrode (FHC, Bowdoinham, ME) placed at the border of corpus callosum separated by ~300–500 μm from the recording electrode. The perfusion saline solution was modified partially as (in mM): 0.5 MgCl_2_, 1 CaCl_2_, 3.5 KCl. Results were obtained using a stimulus intensity to induce 60% of the maximal fEPSP amplitude taken from the input-output curve of each slice. After recording of stable baseline for at least 15 min, LTP induction was performed by applying a high frequency stimulation protocol (HFS, 4 trains, 100 Hz, 1 s duration, separated by 20 s). fEPSP amplitude was monitored for at least 45–60 min after HFS to evaluate LTP. The paired pulse ratio (PPR) paradigm was applied using different inter-pulse intervals and PPR changes were measured as PPR = second fEPSP amplitude/first fEPSP amplitude. For PPR recordings the stimulation intensity was adjusted to induce 60% of the maximal response. All recordings were performed using a Multiclamp 700B amplifier and filtered at 4 kHz and digitized with a Digidata 1440 with pClamp 10 software for data acquisition (Axon, Molecular Devices, LLC, Sunnyvale, CA, USA). Micropipettes were made with borosilicate glass pulled in a P-97 puller (Sutter Instruments, Novato, CA); the recording pipette was filled with the same extracellular solution from the perfusion bath (2–4 MΩ resistance). The perfusion bath was maintained at 25 °C during the recordings (TC-324B Automatic Temperature Controller, Warner Instruments Corporation).

### Conditional Cdk5 knockout mice

Inducible conditional Cdk5 knockout (cKO) was used to study the role of Cdk5 as previously described^33^. Using homologous recombination, exons encoding the Cdk5 catalytic-domain were flanked with *loxP* elements. Homozygous floxed Cdk5 mice were crossed with mice harboring an inducible Cre-ER^T^ recombinase transgene under the control of the prion protein promoter^67^. Cdk5 cKO in homozygous floxed mice carrying the Cre transgene (8 weeks old) was achieved by 14 days administration of 6-hydroxytamoxifen. Homozygous floxed Cdk5 littermates that received vehicle alone injections (without 6-hydroxytamoxifen) served as controls. All the experiments were performed 2–4 weeks after the last injection.

### Selective Cdk5 KO in dorsolateral striatum

Spatially restricted Cdk5 KO was mediated by stereotaxic delivery of a recombinant vector encoding a Cre recombinase-enhanced green fluorescent protein (GFP) fusion under the control of the CMV promoter (AAV2-Cre-GFP, SignaGen Laboratories, Gaithhensburg, MD, USA). Male homozygous fCdk5 mice (7–8 weeks old) were anaesthetized by isoflurane and placed in a stereotaxic frame (David Kopf Instruments, CA) and microsyringes (Hamilton Co, NV) were inserted bilaterally to delivery rAAV (~7 × 10^8^ genome copies, 1 μl/hemisphere) into the dorsolateral striatum (coordinates from bregma at skull surface: +1.5 mm anteroposterior, +2.5 mm lateral, −2.8 mm dorsoventral; 10° angle of entry). Surgical procedures were performed under aseptic conditions after isoflurane anesthesia. Littermate fCdk5 infused with PBS and C57Bl/6 wild-type (WT) mice from Jackson transduced with rAAV2-Cre-GFP served as controls.

### Histology and immunoblotting

Animals were perfused transcardially with PBS solution followed by 4% PFA. Brains were dissected and placed in 4% PFA for 24 h, then block-sectioned and dissected. The 1 cm thick coronal slab which included the injection area (Bregma 1.54 mm) in the center was paraffin-embedded and serially sectioned at 5 μm on a rotary microtome. Slides underwent antigen retrieval (citra solution, BioGenex; 95 °C for 10 min), and were then incubated with 0.3% H_2_O_2_ to remove the endogenous peroxidase activity. Nonspecific antibody binding was blocked with 3% goat serum in 0.3% Triton-X in PBS for 1 h. Primary antibodies included mouse anti-Cdk5 (1:50, PhosphoSolutions), goat anti-GFP (1:400, Thermo scientific) and rabbit anti-NeuN (1:1000, Millipore). For double labeling, primary antibodies were simultaneously incubated (Cdk5/GFP, cdk5/NeuN). For GFP and NeuN, Alexa Fluor 488-conjugated goat anti-rabbit IgG (1:500, Jackson Immunoresearch) were used. For Cdk5, a biotin-conjugated goat anti-mouse IgG (1:2000, Thermo Scientific), followed by streptavidin-HRP and cyanine 3 tyramide (1:50, PerkinElmer), was used. Images were captured using a Zeiss LSM510 Meta confocal laser scanning microscope. Quantitative immunoblot analysis was conducted using previously described standard methodology^68^.

### Behavior

Behavioral testing was conducted 3 weeks after rAAV2-Cre-GFP virus infusion. Locomotor activity was monitored in standard polypropylene cages (15 × 25 cm) equipped with infrared photobeams to monitor activity (Photobeam Analysis Software, San Diego Instruments, San Diego, CA). Locomotor activity was measured over a 2 h period, each day for 6 consecutive days.

To evaluate performance on the accelerating rotarod task, mice were placed on a suspended textured drum covered by a vinyl strip (IITC life Science Inc., Wood Hills, CA). Testing was comprised of 4 trials each day for 4 consecutive days. Subjects were habituated to stay on the non-rotating rod for 2–3 min in the first session. For every subsequent trial, habituation was repeated briefly before rod rotation was initiated. The rod accelerated constantly and uniformly from 4 to 40 rpm over a 300 sec period. Each trial ended when the mouse fell from the rod, which was sensed and recorded by a magnetic switch. Latency to falling was used to derive graphs and analyze data comparatively.

### Statistical analysis

Experimental data to evaluate LTP were analyzed using Student’s *t*-test. One-way ANOVA and Two-way ANOVA for repeated measures (RM) followed by Bonferroni post-hoc test was used where appropriate to analyze the behavioral data. GraphPad Prism 4.0 software was used for graphing and statistical analysis. Data are reported as mean ± s.e.m. Each sample size is indicated. A value of p ≤ 0.05 was considered statistically significant.

## Additional Information

**How to cite this article**: Hernandez, A. *et al*. Cdk5 Modulates Long-Term Synaptic Plasticity and Motor Learning in Dorsolateral Striatum. *Sci. Rep*. **6**, 29812; doi: 10.1038/srep29812 (2016).
